# What we can learn from the dynamics of the 1889 ‘Russian flu’ pandemic for the future trajectory of COVID‐19

**DOI:** 10.1111/1751-7915.13916

**Published:** 2021-08-31

**Authors:** Harald Brüssow

**Affiliations:** ^1^ Department of Biosystems Laboratory of Gene Technology KU Leuven Leuven Belgium

## Abstract

A study of the contemporary medical literature for patient symptoms from the so‐called ‘Russian flu’ pandemic from 1889 revealed clinical observations that resemble COVID‐19 (Brüssow and Brüssow, 2021, *Microb Biotechnol*). If one accepts the hypothesis that this pandemic was a prior coronavirus epidemic, the dynamics of the ‘Russian flu’ from 1889 might give us some ideas about the future trajectory of the COVID‐19 pandemic. The present report compiles and reviews the contemporary data published on the temporal and geographical spread of the ‘Russian flu’, its epidemic wave structure and possible later resurgence. The historical record of past pandemics might thus provide us not with predictions, but ‘retrodictions’ on possible future scenarios for the COVID‐19 pandemic.

## From predictions to retrodictions

What will be the future development of the COVID‐19 pandemic? An answer to this question is of high importance for our societies, affecting private lives as well as future sociological, economic and political developments of entire countries. Projecting future developments is notoriously difficult since it depends on a multitude of parameters while only few are sufficiently well understood to be incorporated into computer simulations (Saad‐Roy *et al*., 2020). Unsurprisingly, computer simulations have a wide margin of uncertainty. Some models predict different trajectories with different degrees of vaccination coverage and maintenance or release of non‐pharmaceutical interventions (Moore *et al*., 2021). Other computer models predicted for COVID‐19 infection waves with distinct periodicity based on different hypotheses on antiviral immunity duration (Kissler *et al*., 2020). When anticipating short‐term immunity duration, the authors predicted annual winter epidemics for SARS‐CoV‐2. With intermediate levels of immunity persistence, epidemics would become biannual. Long‐term immunity would finally lead to extinction of the virus. Other researchers predicted that the transition from epidemic to endemic dynamics will take many years for SARS‐CoV‐2, perhaps decades, but can be accelerated by vaccination (Lavine *et al*., 2021). However, many other factors have an impact on the dynamic development of an epidemic. When a *Nature* feature from August 2020 explored potential future developments for the COVID‐19 pandemic (Scudellari, 2020), the consensus was that nobody dared to make predictions because major parameters impacting the future pandemic course were and still are insufficiently defined. Some projected a major winter peak for 2021 (which occurred), mainly for climate reasons; others suggested that SARS‐CoV‐2 might become endemic with regular annual or biannual winter epidemics. Most researchers expected the virus to stay for a while, and very few postulated that the virus would disappear. The focus of many computer simulations was to explore the effect of control measures, less to study the natural history of the long‐term dynamic development of a pandemic.

Instead of using sophisticated computer models, one can try to project trends from current epidemiological observations into the near future. For example, viral genome sequencing has documented a quite dynamic picture of viral strain replacements during the unfolding of the COVID‐19 pandemic. The original Wuhan strain was worldwide quickly replaced by the D614G mutant (Korber *et al*., 2020). In the UK molecular epidemiologists subsequently observed in late 2020 a surge of the British variant B.1.1.7 (Volz *et al*., 2021), now called alpha variant, and in early summer 2021 the Indian variant B.1.617.2, rebaptized as the delta variant, replaced the previous variants and represents now 90% of the viral strains in diagnostic tests in UK (Lopez Bernal *et al*., 2021). One can thus safely predict that further viral variants will determine the future dynamic of the pandemic. Basic virological reasoning posits that any viral variant showing a higher infectivity will necessarily replace its less infectious forerunners and this process of evolution of increased infectivity will continue as long as the virus is not pushed into extinction by physical containment measures, vaccination campaigns or mutational exhaustion. Recent viral genome analyses demonstrated how SARS‐CoV‐2 variant EU1 spread through Europe in the summer of 2020 without the variant displaying a transmission advantage, because it was pushed by favourable epidemiological settings, namely the resumption of touristic travel to and from Spain (Hodcroft *et al*., 2021). Vaccination campaigns, not reaching herd immunity level, will likely add to this generation of variant viruses by selecting for mutant viruses that escape immune surveillance. Less well established is whether variant coronaviruses will evolve to increased or decreased virulence. So far, the third infection wave in the UK, largely due to the delta variant, is not accompanied by a corresponding mortality wave. The interpretation of this observation is still unclear: while it could indicate reduced clinical virulence of the variant, mortality can still follow with a temporal delay or it might reflect the fact that the clinically most susceptible parts of the British population fell already victim to the previous infection waves. While transmission and thereby also immune evasion are necessary factors for viral survival, virulence increase is not. Some virologists argue that high virulence is a maladaptation of viruses when they cross into a new host to which they are not adapted. According to this school of thought, the most successful virus in evolutionary terms is the one which replicates to high titres in a host without causing significant symptomology, because a lightly affected infected person who maintains active social contact is a better spreader of the virus than a dead host.

However, there is another source of potential insight into the future of the COVID‐19 than computer simulations based on known epidemiological parameters and theoretical reasoning based on evolutionary thinking, namely past experience. One might want to learn from the dynamics of the historic Spanish flu pandemic. This approach has been taken by US scientists who draw on the experience with influenza viruses and general virology from the last century (Telenti *et al*., 2021) However, comparing a pandemic caused by two different viruses, influenza virus and coronavirus, might limit learning lessons for COVID‐19. Epidemics with coronaviruses have occurred in the past (SARS, MERS), but their epidemiological dynamics differed fundamentally from that of COVID‐19 rendering comparisons relatively useless.

Interestingly, clinical observations from the pandemic of 1889 commonly called the Russian flu demonstrated characteristics shared with COVID‐19 (namely multiorgan affections comprising respiratory, intestinal and neurological symptoms; taste and smell loss; long recovery periods; sparing of children) (Brüssow and Brüssow, 2021). There is also some indirect, albeit weak evidence that the pandemic from 1889 might have been caused by a coronavirus, which today is responsible for a certain percentage of seasonal common cold infections in the winter (Vijgen *et al*., 2005). This interpretation would fit with ideas about viral evolutionary trajectories to efficient replication and attenuated virulence and optimists might be tempted to predict a similar fate for SARS‐CoV‐2. Whatever the actual agent causing the pandemic of 1889, the clinical and epidemiological characteristics make the 1889 pandemic the closest we have from the historical record for getting ideas about the dynamics and possible future developments of COVID‐19 not by predictions, but by ‘retrodictions’ from the past.

## Duration of a pandemic

**Fig. 1:**
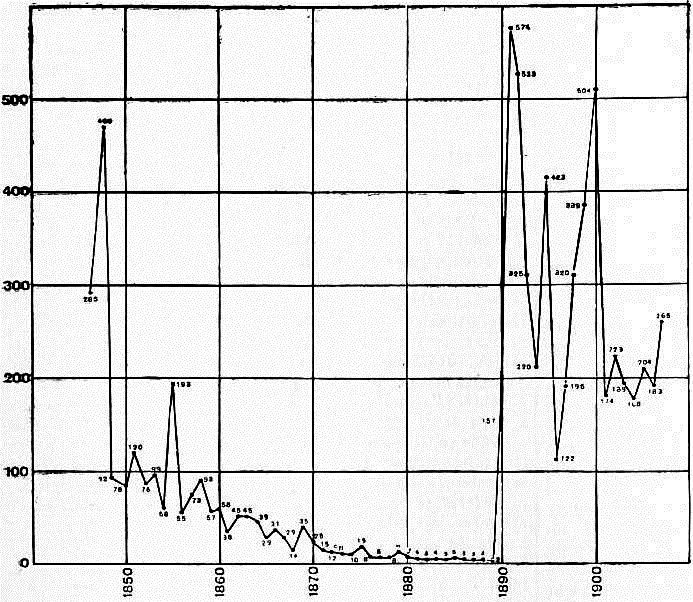
Yearly influenza‐attributed death rates per million inhabitants in England and Wales for the time period between 1840 and 1907 (Source: Encyclopaedia Britannica, 1911).

As we are living through a second year of the COVID‐19 pandemic, one might wonder what the ‘natural’ length of such a pandemic is. If we accept the hypothesis that the clinical symptoms of the 1889 pandemic displayed a COVID‐19‐like clinical character, this historical event might provide an idea about the natural duration of such a pandemic. Good public health data are available for England and Wales with respect to cause‐specific mortality in the 19th century. Fig. 1 displays the death rates directly attributed to what was considered as influenza per million of inhabitants in the UK for the second part of the 19th century. The last epidemic was observed in 1847/8, followed by four decades where influenza deaths were hardly observed. Does this mean that influenza was not any longer circulating in the British population (no seasonal influenza?) and that it needed a reintroduction of a new virus to spark a new epidemic? The Russian flu pandemic arrived in England and Wales in 1890 with a rise to 4523 influenza deaths, followed by further increases to 16 686 and 15 737 influenza deaths in 1891 and 1892 respectively. In 1893 and 1894, these figures decreased, but remained with 9669 and 6625 influenza deaths, respectively, substantially higher than in the pre‐pandemic period (Encyclopaedia Britannica, 1911) (Fig. 1). From these data, one might deduce a protracted 5‐year course for a COVID‐19‐like pandemic, suggesting that COVID‐19 might occupy us well beyond 2022 if the current vaccination campaigns does not change its ‘natural’ trajectory.

## Rapid worldwide spread of the pandemic in 1889/1890

The ongoing COVID‐19 pandemic spread rapidly from China to Europe and then to North and South America spilling over to South Africa and back to Asia with repeated flares in some countries. Is this trajectory a consequence of modern lifestyle characterized by globalization, overpopulation, environmental pollution and accelerated infection transmission by air traffic? While these factors certainly contributed to the spread of the current pandemic, the historical experience from the 1889 pandemic tells us that an epidemic can spread as quickly among a world population of only 1.5 billion people with railroad and ship connections as the quickest means of transportation in a far less connected world than ours (here and in the following, historical data are quoted from Leyden, E., and Guttmann, 1892 or Parsons and Klein, 1893 or Friedrich, 1894, if not indicated otherwise). The first cases of this pandemic were reported in the second half of May 1889 in Bukhara in Central Asia, which belonged then to Russia hence the name ‘Russian flu’. Until June 1889, the outbreak was restricted locally mostly to European inhabitants of Bukhara but then spread rapidly over Turkestan where it infected half of the population. The epidemic took until mid‐October 1889 to reach Tomsk in Siberia. The westward spread of the epidemic was then quick: In early November 1889, it had reached Ukraine and the Black Sea, Moscow and St. Petersburg. End of November, the epidemic was detected in Stockholm, Copenhagen and Berlin. The epidemiologists of the epoch observed that the infection spread along the major railway and shipping lines arriving first at political and trading centres and from there the infection spread star‐like into the surrounding regions. Initially, the infection sprung over large distances without affecting the connecting rural areas. Epidemic dimensions of the outbreak were reported for Berlin in early December 1889; central, western and southern parts of Germany were touched at the end of December 1889. Vienna quickly followed Berlin, and from Vienna, the infection spread over south‐east Europe to Constantinople and reached Greece in January 1890. Paris followed Berlin in the westward spread, and by mid‐January 1890, the whole of France was affected. The Low Countries and Switzerland reported the epidemic in mid‐December 1889. Italy and Spain followed suit, and Morocco was reached in January 1890. High case loads were reported in December 1889 for London, and in January 1890, the whole of Britain went through an epidemic wave. Despite absence of air transport, the infection crossed the Atlantic quickly: the first cases were reported in Boston and New York on 17 December 1889. The spread of the epidemic was likewise rapid over the North American continent showing violent multiorgan clinical manifestations and severe sequels particularly among American Indians and minority ethnic groups in Canada. The epidemic then swept through Central and South America and reached Chile in March 1890. Whether seeded from the initial wave from Turkestan or via Europe, the pandemic arrived in Egypt and Persia in January 1890. British troop transports were reported to have carried the infection to Bombay in March 1890. In April 1890, Calcutta noted an outbreak which touched preferentially coloured people. Southeast Asia was reached in May 1890. Literally, no continent was spared: Africa reported cases in Cape Town already in January 1890 and later along the African coasts, including relatively isolated islands such as Madagascar. In general, the native populations showed more severe infections than the white colonists. Australia and New Zealand reported the epidemic outbreak in mid‐March 1890. Even Greenland and Iceland were touched. The rapid initial spread of COVID‐19 is thus not exceptional and not only linked to the conditions of a connected word in the 21th century, but also occurred with comparable speed in the late 19th century.

## A second wave of the pandemic in 1891

A particularly troubling aspect of the current COVID‐19 pandemic is its occurrence in multiple waves. The periodicity of epidemic recurrence, the length of individual waves and the geographical location of transient epidemic hotspots has puzzled the epidemiologists dealing with the COVID‐19 pandemic and is the reason that the epidemic trajectory has been rather difficult to predict. It seems that we are missing some key determinants of epidemic spread that makes predictions unreliable and gives an erratic aspect to the current pandemic course. Past experience with a COVID‐19‐like pandemic of the ‘Russian flu’ in the 19th century might here again provide some framework and insights. Physicians at that time had expected that the pandemic would run out of steam in late Spring 1890. Therefore, scientists were concerned when a large number of new cases were reported in the summer 1890 on the Azores Islands and when they got news of outbreaks in East Asia with an epidemic in Shanghai / China in October 1890 and a large outbreak in September 1890 over all of Japan. Contemporary epidemiologists suspected that these epidemics were not seeded from the 1890 westward spread returning eastward via Southeast Asia, but might be a recurrence of a prior arrival of the initial infection outbreak from Bukhara. Local epidemic resurge was also observed in several other places, and some cities experienced three or four successive waves, but the outbreaks in the second half of 1890 remained localized with limited spread beyond the surroundings of the affected cities. Localized epidemics were reported in widely scattered areas without obvious geographical connections (e.g. Copenhagen, Silesia, Paris, New York) and, intriguingly, enteric symptoms frequently dominated over respiratory symptoms.

The epidemic situation changed in January 1891 when the epidemic started again in the southern states of the United States, particularly affecting New Orleans. The epidemic then propagated northwards and in March 1891 reached the region between Chicago, Washington DC and New York, where the height of the second wave was seen in early April and finished at the end of April 1891. The disease symptoms corresponded to those seen in the first wave, but showed more neurological symptoms and more severe courses. The next appearance of the epidemic was reported in England where the peak of the second wave was seen between April and May 1891, then shifting to Scotland. The clinical course was frequently severe, and it was observed that regions that were only mildly affected during the first wave in 1890 suffered heavily from infections during the second wave in 1891. The epidemic affected first major cities and then spread radially around neighbouring areas. The pace of epidemic spread in England was slower in the second wave than during the first wave. Reinfections were observed in 5% of persons already infected during the first wave. Epidemiological characteristics of the infections changed between the two waves: in the second wave, more children and more privileged social strata were affected than in the first wave. The epidemic spread then showed two trajectories: one northern route reaching out to Norway (arriving there in mid‐May 1891), Sweden (end of May 1891) and Denmark (where many cases, but low mortality was observed); and a southern propagation wave reaching Portugal and Spain in August/September 1891, where the patients showed many neurological symptoms, but only low mortality. From there, the epidemic progressed to southern France (October 1891) and reached Paris in November 1891. The northern epidemic route subsequently progressed to St. Petersburg from where it spread southward to Ukraine (December 1891) and then Constantinople (January 1892) and westward to Poland and Germany. Germany was thus re‐exposed to the second wave from two sides: from France in the west and from Poland in the east. Berlin experienced an increase in mortality in November 1891 mostly in persons older than 60 years. From Germany, the epidemic then spread to Italy where Milano, Torino, Venice and Genoa suffered high mortality increases. The peak of new cases in Italy was seen between January and March 1892. Contemporary epidemiologists noted that the trajectory of the second wave (West to East) was just the reverse of the spread from the first wave (East to West).

## Third and later waves

At the end of December 1891, physicians observed another surge of the epidemic in Scandinavia from where it spread to The Netherlands and Ireland (which was strangely not touched by the May 1891 epidemic in England), then back to the United States and to Australia. In August 1892, a late large outbreak was reported in Peru. Cases were still reported up to December 1892 and January 1893 in Germany, Belgium, Spain, Russia and the United States. The last phase of the pandemic was not characterized by directed spread of the epidemic along defined trajectories, but by the revival of the infection foci in geographically scattered places. Overall, the pandemic showed a mixture of directed geographical spread parallel with human movements combined with an element of unpredictability, suggesting complex causal relationships or even some stochastic elements. In analogy with the current COVID‐19 pandemic, the waves observed in the Russian flu pandemic might represent the appearance of variant viruses.

## Epidemiological inferences

The source for renewed infection waves was at the time controversially discussed. Scientists invoked human and animal reservoirs or physical persistence of the infectious agent in the abiotic environment. However, for the first two epidemic waves, there was a consensus about a direct person‐to‐person infection transmission route and that the speed of propagation was never more rapid than that of the most rapid means of human transport. This observation excluded transport by wind. Air transport was still popular at the time when the germ theory had not yet fully replaced the miasma theory (poisonous exhalation of the soil carried away by winds) of epidemics (see the name of malaria, literally bad air). During the first wave, the expansion of the epidemic was along the major railway and shipping lines from capital to capital, followed by capital to surrounding periphery spread. Crowding and insufficient aeration of rooms were at the time defined as risk factors, while sunlight and humidity were recognized as negatively affecting the viability of the agent, thus reducing transmission. The agent was suspected to represent a bacterial pathogen, while a German report spoke already of a virus, then, however, meaning a toxin. The physicians postulated that the agent could gain or lose virulence and change its clinical phenotype which might explain the variation in clinical severity seen between different places or at different times of the epidemic at a given place. While some authors alluded to the hypothesis of two different, sequential epidemics with two different agents (this would potentially reconcile the two competing hypotheses of an influenza virus or a coronavirus causing the 1889 pandemic), the general consensus was that of a single pandemic over a 3‐ to 5‐year period. Interaction with other infectious diseases was observed, rendering their clinical expression more severe.

## How many individual waves occurred at specific places and what was the length of their individual duration?

Detailed influenza mortality data are only available for a few cities. In London, deaths attributed to influenza increased substantially over a 4‐week period starting in mid‐January 1890 with peak death counts over two weeks which then gradually levelled off over the next two months to remain at low values over the rest of the year. A second peak of influenza mortality in London was observed with even higher death numbers over a 8‐week period between May and June 1891, again levelling off to low values for the rest of the year. A third mortality peak over 5 weeks was noted in January/February 1892, again followed by low influenza mortality values for the rest of the year. Different English cities all showed comparable dynamics with temporally relatively sharply defined three mortality waves. Three clearly defined influenza mortality waves were also observed in geographically distant places where reliable census data were gathered, such as in the United States. For example, in the US state of Indiana, mortality peaks were noted for a first wave in January to March 1890, for a second wave in March to April 1891 and a much larger peak for a third wave between December 1891 and February 1892. No further waves were seen in 1893. While the British and US influenza deaths were relatively synchronized, this was not the case for other places (Ewing, 2019). For example, Copenhagen also experienced three successive waves, but with distinct dynamics: the first wave extended over a longer time period from December 1889 to July 1890; a small local outbreak occurred in October 1890, and a final epidemic wave was observed in July 1891. In contrast, a single infection wave was observed for example in Madrid where the evaluation of a governmental official newspaper noted between 1888 and 1892 a sharp mortality increase for December 1889 with less than a month duration. A second mortality peak in December 1890 was only slightly higher than that observed during December 1888 in the pre‐pandemic period (Ramiro *et al*., 2018). The long duration of the first epidemic wave in Copenhagen was an exception: in St. Petersburg, Vienna, Berlin, Brussels, Paris and New York, individual epidemic waves showed sharp excess mortality peaks of only few weeks duration (Valleron *et al*., 2010).

Data on morbidity are also available for Sweden where questionaries were answered by 400 doctors treating people in 69 localities (Skog *et al*., 2008, 2014). A sudden increase of infected persons was reported for Sweden in mid‐December 1889, the numbers of infected people crossed 100 000 in mid‐December, reached a peak with more than 700 000 cases in the first week of January 1890 and decreased to pre‐epidemic levels in early March 1890. However, when analysed at a district level, the epidemics were of shorter duration covering just 1 or 2 and at maximum 3 weeks.

## Attack rates, case fatality ratios and R values

A Swedish survey calculated an attack rate of 60% for the entire population with no major difference between males and females (Skog *et al*., 2008). Geographically, the entry point was Stockholm and then the epidemic spread along the railway network. In the first week of December 1889, 12 out of 13 affected places had a railway station. In the third week of December 1889, 82% of places with a railway station were affected, compared with only 47% of places lacking a railway station. When the epidemic regressed in Southern Sweden, a second infection focus around the seaport Lulea in Northern Sweden was observed. The data supported the importance of person‐to‐person contact for disease transmission facilitated by transportation. In a similarly organized, but larger survey in Germany, a wide variation in attack rates was observed ranging from 20% to 80%. The German physicians estimated an average attack rate of 60%, but were unable to correlate epidemiological factors with the reported variation in attack rates (Leyden and Guttmann, 1892). Berlin for example noted a 33% attack rate, while Nuremberg reported one of 67%. The remote and sparsely populated North Sea island Helgoland experienced a 50% attack rate. Some areas in Southern Europe reported even higher attack rates of 70% for some areas in Southern Italy and up to 90% for Portugal. (Encyclopaedia Britannica, 1911) The evaluation of statistical reports from the 1889–1890 for Switzerland (Schmid, 1895) and Sweden showed relatively uniform attack rates of 60% across all age groups except for infants which showed clinical attack rates of less than 20% in Switzerland (Valtat *et al*., 2011).

Mortality from influenza by age group was J‐shaped (moderately elevated in the very young, going through a minimum in the young and then increasing steeply with advancing age) in Switzerland, but excess mortality was not observed in younger age groups and the highest mortality burden was experienced by >60 year old people. In Switzerland, the high attack rate was not mirrored by a correspondingly high mortality rate, suggesting a low case fatality ratio (Valtat *et al*., 2011). Estimates for the case fatality ratio (CFR) were put together by French scientists who based their calculation on data reported for the French, British and German armies, as well as on surveys performed in seven Swiss cities. The CFR ranged from 0.1% to 0.28% (Valleron *et al*., 2010). Substantial differences were seen between different temporal phases of the pandemic and between different epidemiological settings, making CFR calculations a difficult task. The case numbers in England were much higher in 1890 than in 1891; for example, the number of persons treated at the London Middlesex Hospital in the two month‐winter epidemic of 1890 was 1279; in the three month‐spring epidemic of 1891, it was only 726. However, in 1891, mortality rates were 3.7‐fold higher in London and 5.5‐fold higher in English cities than in the corresponding places in 1890. Similar mortality increases were seen for smaller English towns and rural areas, when data from 1891 and 1890 were compared. Contemporary physicians attributed this mortality difference to a changed onset of disease: it was sudden in 1890 such that the patients immediately sought bedrest (the only valid treatment mode at the time) while it was insidious in 1891 such that bedrest was sought later, causing frequently protracted and complicated disease forms. On the whole, rural districts in England showed a higher death‐rate than towns, and small towns a higher one than large ones in both years (Encyclopaedia Britannica, 1911).

Modern French researchers calculated the median basic reproduction number (R_0_) to 2.1 (range 1.9 to 2.4) when evaluating data from 96 cities from the 1889 pandemic, using a discrete time Susceptible–Exposed–Infected–Removed (SEIR) model (Valleron *et al*., 2010). The relative mortality increase compared with baseline mortality and R_0_ were both negatively correlated with latitude in Europe, that is the mortality burden was higher in the southern region of Europe than in the northern parts. However, such a latitude correlation was not observed for North America. R_0_ was not statistically linked to the population size of the investigated cities. Interestingly, the R_0_ values for these cities were correlated when the 1889 and 1918 pandemic were compared, indicating that pandemics that differ substantially in basic characteristics (e.g. the 1918 Spanish flu had a W‐shaped age mortality curve compared with the J‐shaped age mortality curve of 1889) display nevertheless common characteristics that allow some comparisons and possibly some pre‐ or retrodictions between different pandemics (Valleron *et al*., 2010).

## Newspaper reports

The evaluation of historical reports from newspapers as well as from scientific and medical journals has been facilitated by the digital access to these reports. For example, from the Austrian Newspapers Online (ANNO) repository, more than 600 news articles about the Russian flu were retrieved from 42 newspapers. Likewise, papers from medical journals such as *The Lancet* can now be searched online back to 1823 (The Lancet archives (upenn.edu)); also, the *British Medical Journal* covers medical reports back to 1840 in searchable archives (The BMJ: browse by volume/issue, medical specialty or clinical topic | The BMJ). For example, Polish historians of medicine systematically evaluated the daily newspapers from Poznan which reported on the spread of disease in Europe quoting newspapers from Paris (Le Temps, Le Matin), Berlin (Vossische Zeitung) and London (The Times) (Kempińska‐Mirosławska and Woźniak‐Kosek, 2013). Hospitals in many capitals were overburdened; in France, the military had to place tents for the diseased in the hospital gardens. The health crisis was accentuated by the fact that many doctors and nurses contracted the disease. The high attack rate can also be read from the closure of schools and universities because a large part of the teaching staff fell ill. Post services and fire brigades suffered interruptions of their services, and factories had to be closed because a large part of the workers contracted the disease. Statistical reports quoted by the newspapers noted that mortality rates had increased by 30% compared to the same time period of the pre‐pandemic year. Funeral homes were overwhelmed and asked for simplified funeral rites. In Paris, funerals were as numerous as during the siege of Paris in 1870. In Madrid, funerals were done at nighttime to avoid panic in the population (Kempińska‐Mirosławska and Woźniak‐Kosek, 2013).

## Recurrence of the pandemic?

In 1895, the influenza‐associated deaths in England and Wales rose again to 12 880, followed by a drop in 1896 to 3753 influenza deaths. However, the influenza mortality increased again in the next year to 6088 deaths and then showed another broad peak in 1898, 1899 and 1900 with 10 405, 12 417 and 16 245 deaths respectively. These numbers define another three‐year course with elevated death counts (Encyclopaedia Britannica, 1911) (Fig. 1) and ask the question whether the 1898–1900 epidemic is a recurrence of the 1889 pandemic?

Ten years ago, British scientists wrote that the outbreak of 1900 attracted little attention at the time, and its impact was noted by few modern reviewers. However, an increase in cases and spread of influenza at that time was observed in London, Australia and North America, and indirect data indicated that approximately 80% of persons were infected. The lack of clinical data suggests that the outbreak was mild in severity (Potter and Jennings, 2011).

When searching *The Lancet* archive from 1900, a different picture emerges: An editorial from 13 January 1900 (vol. 1, p. 107) states that England is in the middle of an influenza epidemic and encourages the citizens to stay at home when early disease symptoms become manifest to contain a further spread of the epidemic. Participation at public gatherings were strongly discouraged and the editorial mentions that also private parties should be avoided. If one looks through the obituary sections for noted physicians of the *BMJ* in 1900, it is striking that practically all died from influenza during the time period between January and April 1900. British physicians and pathologists summarized the clinical aspects of the influenza cases in a *Lancet* report from 4 August 1900 (vol. 2, pp. 362–363) of the 1900 outbreak. They distinguished four forms of influenza, which attacks (i) the mucous membranes; (ii) the gastrointestinal tract; (iii) the heart; and (iv) the nervous system. Dyspnoea (shortness of breath) and thrombi particularly in the brain were noted. Based on these symptoms, they suspected a reappearance of the 1889–1890 epidemic after 10 years. The meeting report also mentions the observation of anosmia (loss of smell), diabetes after influenza infections and post‐influenza sequels of nervous disorder. This symptom complex is indeed similar to the clinical symptoms described for influenza patients from 1889 to 1890 in comprehensive British and German reports (Brüssow and Brüssow, 2021). Since these observations resemble more a COVID‐19 related disease than classical influenza, we must consider the possibility of a coronavirus‐induced pandemic in 1889–1890 and a resurgence of this pandemic 10 years later, peaking in 1900. This was also the thought of physicians describing cases in 1898 when the resurgence started. Containment of the epidemic by patient isolation was not very efficient because physicians noted that infection was transmitted before symptoms were recognized in the patients and that the patients showed such a variety of symptoms that their identification was difficult. Many subjects that were infected in 1890 suffered a reinfection in 1898 (Anonymous, 1900a). In 1899, large establishments – commercial, educational, religious, including the House of Commons – were seriously crippled by the ravages of the disease, not only on account of the imperfect provision for ventilation, but also on account of the large gathering of persons. Infection was frequently followed by pneumonia, often of a severe and dangerous type. The obituary lists of the daily papers contained a dismal total of mortality ascribed to ‘pneumonia’ (Anonymous, 1899). In England of 1899 with a population of 30 million, at least 220,830 persons were affected by influenza in the course of a year, 31,950 of these persons died from its effects. People living under good hygienic conditions and not mixing up with others were relatively spared from the infection (Anonymous, 1900b). In 1899, people older than 60 years showed a mortality increase of 14% compared to the pre‐pandemic period (Anonymous, 1900c). Physicians noted in influenza patients from 1900 that the nervous system was almost always affected, sometimes with a widespread and bizarre distribution of symptoms. Neurasthenia, that is mental exhaustion with headaches, insomnia (sleeplessness), and irritability as a consequence of depression or emotional stress, belonged to the sequels of infections in many patients (Anonymous, 1900d). Other physicians noted that the gastrointestinal form of the disease from this epidemic may closely simulate enteric fever (Anonymous, 1900e).

If 1898 to 1900 was a reappearance of the 1889 infectious agent, it was its last appearance. For the next 18 years, no excess mortality peaks were observed. Infectious diseases mortality in industrialized countries showed a constant albeit small decrease over the next years until interrupted by a sharp mortality increase in 1918–19 caused by the Spanish flu (Feigenbaum *et al*., 2019), which was definitively caused by an influenza virus.

## Outlook

Whether the pandemic starting in 1889 can serve as a paradigm for the future evolution of the COVID‐19 pandemic hinges on a number of assumptions. It is so far conjectural that the 1889 pandemic was caused by a coronavirus infection. That the disease caused by the 1889 pandemic shares some characteristics with COVID‐19 with respect to clinical symptoms and epidemiology is better supported by contemporary reports (Brüssow and Brüssow, 2021). However, even if one accepts this as a working hypothesis, it is by no means clear whether an epidemic with similar basic characteristics will be a replay of one which occurred 140 years ago. There are too many differences that distinguish the current epidemic situation from that of the final years of the 19th century. Just to name a few: In contrast to its widespread use during the Spanish flu pandemic of 1918, face masks were not used during the 1889 pandemic. In fact, the first medical use of face masks as anti‐infection measure was only introduced in1897 by P. Berger, a surgeon working in Paris. Public health measures during the 1889 pandemic consisted mainly of school closures and hygiene advice (handwashing). Factory and public services experienced closures not as a containment measure, but because too many people fell ill. Quarantine measures were not used during the 1889–1890 pandemic. Country‐wide lockdowns were not heard of in the late 19th century. Vaccines were not available against respiratory infections – infections were thus transmitted relatively unrestrained. Intensive care medicine was in 1889 practically non‐existent, and the best medical advice of the time was early bedrest and antipyretics. These differences will certainly modify the course of a pandemic. However, in view of these differences, it is astonishing how similar the pandemics from 1889 and 2019 behaved in their rapid worldwide spread, their death toll and the waxing and waning of case numbers creating distinct waves of infection.

Of course we are all eager to learn when and how the COVID‐19 pandemic will end. If the data from the end of the 19th century are an indication, COVID‐19 may occupy us for a decade in multiple infection waves without much clinical attenuation if not stopped by vaccination programmes that achieve herd immunity or breakthroughs in drug development which make COVID‐19 a treatable disease with low mortality. US researchers using influenza pandemics as a model for the future development of COVID‐19 considered three scenarios: (i) ongoing severe disease manifestations combined with high levels of infected individuals which foster further evolution of the virus; (ii) a transition to an epidemic seasonal disease like influenza with an annual mortality burden of 250,000 to 500,000 people globally; (iii) or an endemic disease similar to other human coronavirus infection that have a much lower disease impact than influenza (Telenti *et al*., 2021). With vaccine hesitancy or explicit opposition to vaccination in a substantial part of the populations (which will make herd immunity an illusion, particularly in view of the evolution of ever more transmissible virus variants) and the unwillingness of political authorities to make vaccination obligatory, it is unlikely that vaccination will stop the pandemic soon. Humans might also have transmitted the infection to domesticated and wild animals which might reintroduce virus and viral variants into the human population. Efficient drugs are therefore needed to make COVID‐19 a treatable disease. With efficient drugs and vaccines at hand, a re‐opening of our societies can be envisioned without risking substantial human mortality. Without these options, the pandemic of 1889 might have taken nearly a decade to subside.

## Funding Information

No funding information provided.

## Conflicts of interest

None declared.
